# The multiverse of CD46 and oncologic interactions

**DOI:** 10.1172/JCI188355

**Published:** 2025-05-01

**Authors:** M. Kathryn Liszewski, John P. Atkinson

**Affiliations:** Division of Rheumatology, Department of Medicine, Washington University School of Medicine, St. Louis, Missouri, USA.

## Abstract

Initially identified as a regulator of complement activation on host cells, the known roles of CD46 (membrane cofactor protein [MCP]) have expanded. We now know that this ancient molecule is expressed on almost all nucleated cells as a family of four predominant isoforms. It also is involved in human reproduction, modulation of T cell activation and immunoinflammatory effector functions, autophagy, and the newly identified intracellular complement system (complosome). CD46 is also known as a “pathogen” magnet, being a port of entry for at least seven bacteria and five viruses. Moreover, CD46 has recently emerged as a key player in cancer biology. Numerous studies provide evidence of the association among elevated CD46 expression, malignant transformation, and metastasizing potential. These features, along with its roles as pathogen receptor, have made CD46 a target for cancer therapeutics. Thus, modified viral vectors (such as strains of adenovirus and measles virus) targeting CD46 currently are being exploited against a wide range of cancers. Another oncologic treatment utilizes a CD46-targeting human mAb as an antibody-drug conjugate. Herein, we review CD46 and its “multiverse” of cancer interactions.

## Introduction

CD46 (also called membrane cofactor protein [MCP]) plays multifaceted roles in health and disease. It was initially identified as a C3b-binding protein utilizing C3b and C3(H_2_O) affinity column chromatography (1) and was subsequently demonstrated to serve as a regulator of complement activation on host cells (refs. 1, 2 and reviewed in ref. 3). We now know that CD46 is expressed on most cells, where it helps mitigate the activation of the complement system in order to focus and limit complement attack to invading pathogens and damaged tissue (3, 4).

CD46 also is becoming recognized as a potential tumor target or prognostic indicator because complement (and CD46) expression is often dysregulated (i.e., increased) during tumorigenesis (5, 6). Clinical and experimental data support an association between higher expression and malignant transformation as well as potential to metastasize. To better understand CD46 as a player in cancer biology, it is helpful to have a more complete background (including structural and functional profiles) of this multitalented protein.

### Complement and CD46.

Complement activation leads to the rapid identification and destruction of invading pathogens (7). The system also is a key mediator of local inflammation and a director of adaptive immunity (8). Because of the complement system’s proinflammatory, immune-enhancing, and cell/tissue-damaging capabilities, nearly half of complement components function in its regulation/inhibition. Indeed, this provides the basis for recognition of “self” from “nonself” in that self cells, bearing complement control proteins, are protected while nonself (e.g., bacteria and damaged cells) are attacked. Complement’s regulators are expressed in the fluid phase (plasma) and on cell membranes. One such group of genetically, structurally, and functionally related membrane and plasma glycoproteins is the regulators of complement activation (RCA) gene cluster that lies on the long arm of chromosome 1 (1q32) (9, 10). Tasked with controlling the activation of complement’s central components, C3 and C4, this family includes decay accelerating factor (DAF, also known as CD55), complement receptors 1 (CD35) and 2 (CD21), C4b binding protein (C4BP), factor H (FH), and CD46. We will focus on CD46 for this Review.

### Identification and cloning.

**Figure 1:**
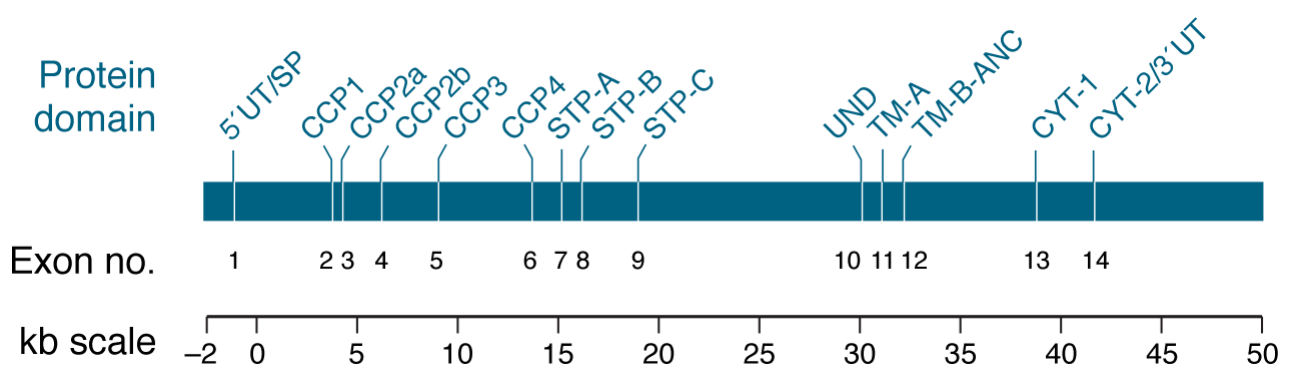
Genomic organization of *CD46*. The alternatively spliced *CD46* gene lies at 1q32 and consists of 14 exons and 13 introns for a minimum length of approximately 43 kb. The protein domains, exon number, and approximate sizes (in kb) are shown. Exons are represented by vertical lines on the protein domain, and exon lengths are not to scale. The protein domains include 5′ untranslated area and signal peptide (5′UT/SP); complement control protein modules (CCP1, CCP2a, CCP2b, CCP3, and CCP4 modules); alternatively spliced exons coding for segments enriched in serines, threonines, and prolines (STP-A, -B, and -C); segment of undefined function (UND); two exons that code for the transmembrane domain (TM), TM-A and TM-B-ANC, which also codes for the intracytoplasmic anchor (ANC); and alternatively spliced cytoplasmic tail 1 (CYT-1) and cytoplasmic tail 2 and 3′ untranslated region (CYT-2/3′UT). Adapted with permission from *Annual Review of Immunology* (11).

**Figure 2:**
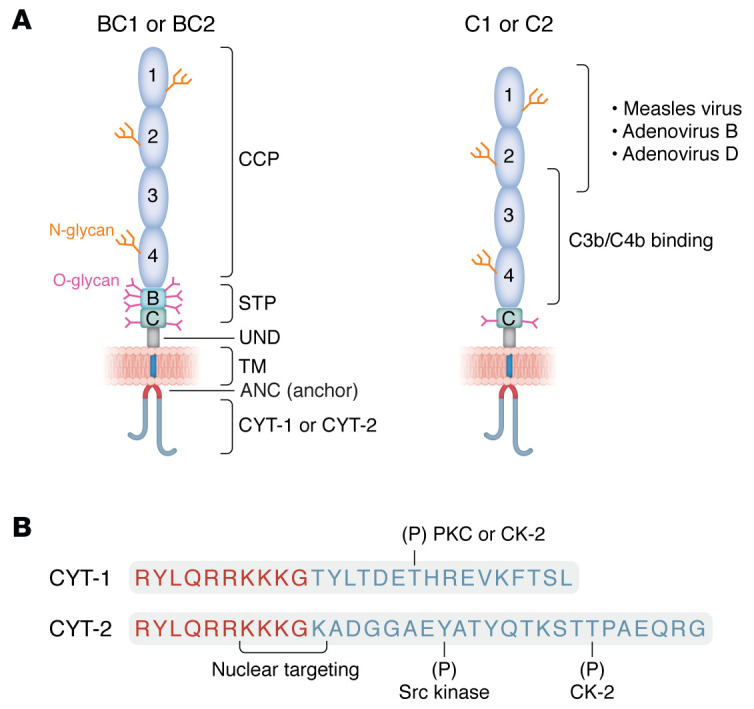
CD46 protein structure and cytoplasmic domain detail. CD46 is expressed on nearly all cells of the body, with the notable exception of erythrocytes, as an alternatively spliced complement regulatory protein that also plays roles in several other processes, such as reproduction and cellular metabolism. It is also a receptor or adherence factor for at least 12 pathogens. These include measles virus (vaccine strain) and some species of adenoviruses that are utilized in cancer targeting therapeutics (see text). (**A**) CD46 structure is dominated by the presence of four CCP repeats of approximately 60 aa each. CCP2–CCP4 are the primary sites for C3b/C4b regulatory function. CCP1, CCP2, and CCP4 possess N-glycans. Next is the alternatively spliced STP segment that is a site for *O*-glycosylation. This is followed by a short segment (13 aa) of undefined function (UND) and the TM and intracytoplasmic anchor. Alternative splicing also produces two separate cytoplasmic tails (CYT-1 and CYT-2) with distinct signaling motifs. Four common isoforms are coexpressed to variable extents on most cells and are termed BC1, BC2, C1, and C2. (**B**) Aa sequence of the intracytoplasmic anchor (red) with CYT-1 or CYT-2. Potential phosphorylation (P) and nuclear localization signaling sites are indicated. Adapted with permission from *Current Opinion in Immunology* (4). Most abbreviations are defined in the legend for Figure 1. CK-2, casein kinase II.

While earlier publications refer to CD46 as MCP or gp45-70 (reflecting its functions and/or electrophoretic profile), it is more often now referred to as CD46 to avoid confusion with the later discovered monocyte chemotaxis protein that is referred to by the symbol MCP-1. CD46 was initially identified in a search for C3b- and C4b-binding complement receptor and regulatory proteins (3). Studies determined that it served as a cofactor for the plasma serine protease factor I (FI) to cleave and inactivate C3b and C4b that deposit on host cells. Interestingly, its unusual electrophoretic profile consists of several variably expressed species with relative molecular masses ranging from 45 to 70 kDa. This characteristic was subsequently explained by its cloning and genomic organization revealing that CD46 was alternatively spliced from a single gene of approximately 43 kb, consisting of 14 exons and 13 introns (11) (Figures 1 and 2). Thus, CD46 is coexpressed as a family of four predominant isoforms in varying proportions on most all cells, except erythrocytes (4).

Each of the isoforms shares an identical amino terminus consisting of four approximately 60–70 aa repeating units called complement control protein (CCP) modules (also known as sushi domains or short consensus repeats [SCRs]) (Figure 2). The isoforms also share an alternatively spliced domain enriched in serines, threonines, and prolines (STP), a site for *O*-linked glycosylation. Three short exons code for peptides of STP-A (15 aa), -B (15 aa), and -C (14 aa). The more common isoforms exclude A and contain B and C or C alone. This region is followed by a juxtamembranous segment (12 aa) of undefined function, a hydrophobic transmembrane domain (24 aa), and a charged intracytoplasmic anchor (10 aa). The carboxyl terminus contains one of two alternatively spliced, nonhomologous cytoplasmic tails, each of which bears distinct signaling motifs. CYT-1 consists of 16 aa, while CYT-2 contains 23 aa. Thus, CD46 isoforms are termed BC1, BC2, C1, and C2, reflecting the variations in their STP and cytoplasmic tail domains. The glycosylation differences in the STP domain largely account for its broad or two-band electrophoretic profile; that is, the higher-molecular-weight species contain BC1 and/or BC2 isoforms whereas the less glycosylated lower-molecular-weight isoforms consist of C1 and/or C2.

### Functional profile.

For its role as a regulator of complement activation, CD46 binds C3b or C4b after their deposition on a host cell. Note also that CD46 can be shed from cells, yet retain binding to C3b as well as the C3b-like product, C3(H_2_O), that is generated on a low but continuous basis (tickover) (12, 13). These activation products are inactivated as CD46 serves as a cofactor for their cleavage by FI. CD46 is particularly potent in guarding against activation of the self-amplifying alternative pathway (14, 15), albeit BC isoforms show enhanced protection against the classical pathway relative to the C isoforms (15, 16).

Since its identification nearly 40 years ago as a C3b-binding protein, the functional profile of CD46 has expanded (3, 17). Surprisingly, it plays a role in fertilization, in that the C2 isoform is solely expressed on the inner acrosomal membrane of human spermatozoa, where it may assist oocytes with the interaction during fertilization or protect spermatozoa from C3b deposition during penetration (18, 19). The two cytoplasmic tails of CD46 differentially mediate intracellular signaling that affects cell behavior. For example, *Neisseria* infection of epithelial cells (via CD46) leads to phosphorylation of CYT-2 by the Src kinase c-Yes, a process that may be important for *Neisseria* attachment and cytoskeletal rearrangements (17, 20). Furthermore, in epithelial cells, CD46 regulates autophagy during pathogen invasion. This is mediated by CYT-1 that is linked to the autophagosome via interaction of its C-terminal tetrapeptide (FTSL) with the scaffold protein GOPC (21, 22). CYT-1 also binds the scaffold protein DLG to mediate epithelial cell polarization (23).

The signaling capabilities of CD46 have been more extensively studied in T cells, where CD46 plays a key role in cell regulation (24, 25). Each cytoplasmic domain differentially mediates proliferation and effector functioning. Thus, CYT-1 mediates cell activation and cytokine production (26, 27) while CYT-2 directs contraction of both processes (27). CD46 cytoplasmic domain switching links Th1 cell activation and then contraction to a pathway for metabolic reprogramming (28). CD46 also provides costimulatory signals for optimal cytotoxic CD8^+^ T cell development by augmenting nutrient-influx and fatty acid synthesis (29, 30). Furthermore, an in-depth investigation regarding T cell activation led to the discovery of an intracellular complement system (complosome) (28, 31), resulting in an explosion of new studies investigating and expanding on these findings (32, 33).

### Pathogen magnet.

CD46 has been described as a “pathogen magnet” since it is a target of at least 12 human pathogens (4, 34–39). This group includes five viruses and seven bacteria: viruses include multiple species of adenoviruses (AdV types B and D) (36, 40, 41), measles virus (MV; the vaccine strain or laboratory-adapted strains) (34, 35), herpesvirus 6A (42), and CMV (4, 43); bacteria include *Streptococcus pyogenes* (44), *Neisseria gonorrhea* (45), *Neisseria meningitides* (45), *E*. *coli* (46), *Klebsiella pneumoniae* (47*)*, *Fusobacterium nucleatum* (4, 48), and *Edwardsiella tarda* (49).

Interestingly, pathogens target various CD46 domains for attachment and entry. Gaggar et al. determined that Ad35 binds to CCPs1-2 and that it competes with MV for binding (36). Furthermore, following engagement, CD46 can be either internalized or shed. The appeal of CD46 to pathogens may not only encompass its widespread expression but may also include its immunomodulatory signaling capabilities (4). For example, MV (vaccine strain) downregulates expression of IL-12 by monocytes via its binding to CD46 (50, 51). As will become evident through the discussion in this Review, this connection of specific viruses with CD46 will have important implications for anticancer therapeutics.

## CD46 and cancer

While the activation of complement can be considered a critical survival mechanism, its role in cancer is a double-edged sword; i.e., it can assist with the killing of tumor cells, but it also can promote tumor growth, inflammation, and immunosuppression. It has been suggested that cancers possess their own unique interplay of complement components governed by both the tumor microenvironment and the tumor cell itself (52–57).

While it might be expected that activation of complement on the surface of cancer cells should serve to inhibit cancer progression via opsonization or through membrane-attack complex–mediated membrane perturbation, this is not always the case. A number of studies have demonstrated that activation of complement can also serve to promote cancer progression particularly via the actions of generated anaphylatoxins (C3a, C5a) and their impact on the tumor microenvironment as well as by instigating activation of immunoevasive responses and pathways (52, 58).

**Figure 3:**
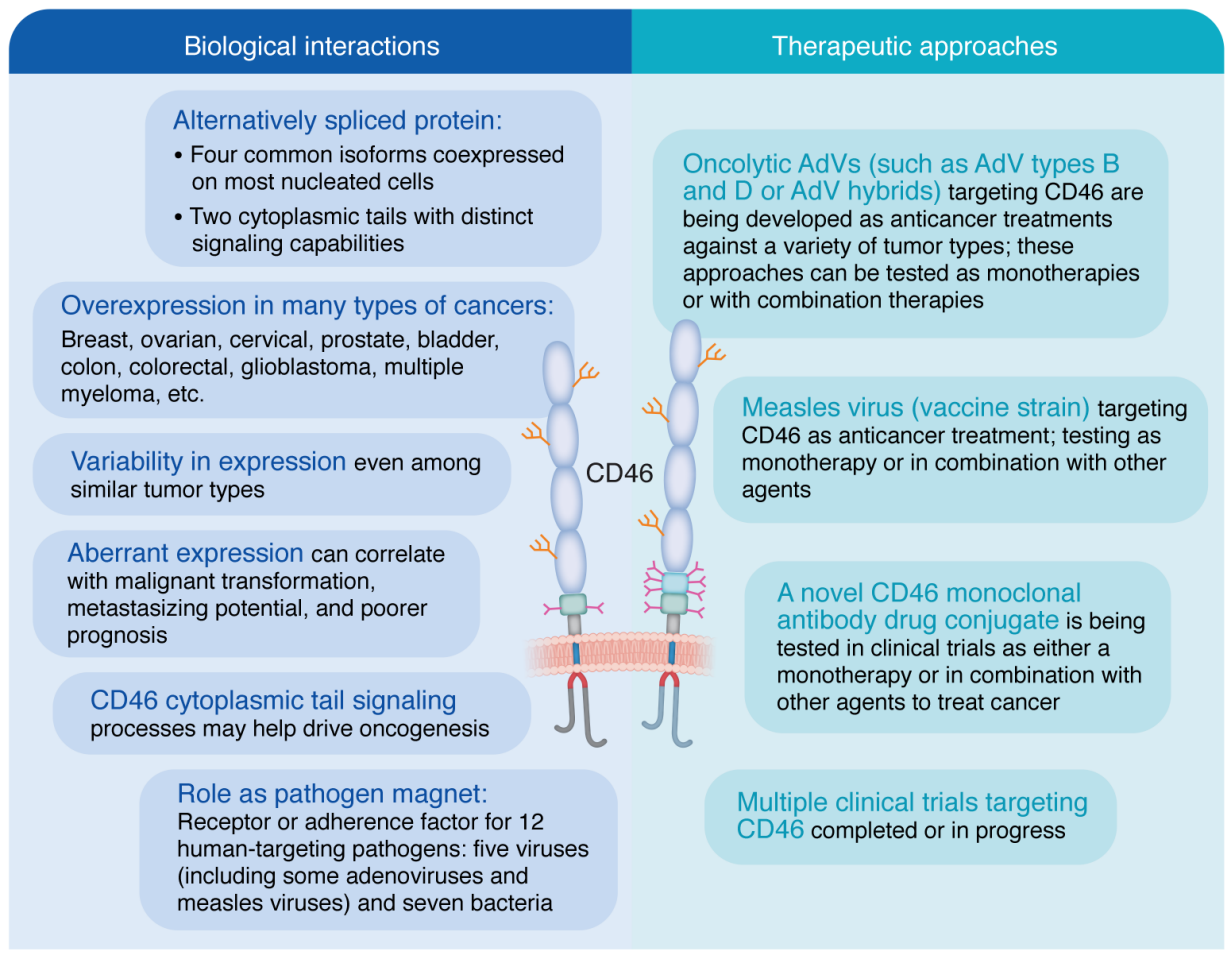
The multiverse of CD46 and oncologic interactions. CD46 plays multifaceted roles in complement regulation and cell biology, including functioning as a tumor driver as well as a target for anticancer therapeutics. AdV, adenovirus.

Thus, increased CD46 expression on malignant cells could be considered an immune evasion mechanism to prevent complement activation to benefit the tumor cell (Figure 3). Indeed, its overexpression in malignancies was identified soon after its discovery (59, 60). Since then many studies have documented CD46 upregulation in multiple myeloma (MM) and in a host of solid tumors, including ovarian, breast, cervical, colorectal, prostate, bladder, and others (5, 6). Such aberrant expression may also correlate with malignant transformation and metastasizing potential, as discussed below.

For example, in MM (a malignancy of the B cell lineage), CD46 expression in patient myeloma cells was found to be increased up to 14-fold as a result of the genomic amplification of a segment on the CD46-containing segment of chromosome 1q (61). In hepatocellular carcinoma (HCC), expression was increased 6-fold relative to normal cells and occurred as an early event (62). Additionally, high concentrations of soluble CD46 (that remain functional) have been observed in the sera of patients with cancer (63–65). Other clinical and experimental data from a variety of tumors correlated CD46 expression with malignant transformation and metastasizing potential (reviewed in refs. 5, 6). In breast, ovarian, cervical, and hepatocellular cancers (as well as MM discussed above), increased CD46 expression was associated with poorer survival/prognosis (65–68).

Several investigations have sought possible mechanisms examining the effects of CD46 in facilitating cancer invasion and/or metastatic potential. In a study utilizing bladder cancer cell lines, CD46 overexpression enhanced the upregulation of MMP9 to trigger phosphorylation of p38 MAPK and PKB and to promote increased activity of activator protein 1 (AP-1) activity via c-Jun (69). This indicated that CD46 facilitates bladder cancer cell migration and invasion via MMP9 expression. In another study, using the HepG2 cell line and HCC-containing tissues, Lu et al. utilized bioinformatics to identify let-7b and miR-17 microRNAs as targets of CD46 signaling (68). The expression levels of both negatively correlated with CD46, indicating that CD46 may play an important role in HCC carcinogenesis via microRNAs. Aberrant signaling processes may also drive oncogenesis. Buettner et al. correlated the binding of activated STAT3 to overexpression of CD46 mRNA and protein (70). STAT3 can be overactivated in a variety of cancers. Using microarray gene expression profiling, STAT3 was observed to bind two sites on the CD46 promoter to induce expression. In Buettner et al.’s report, this upregulation protected cancer cells from complement-dependent cytotoxicity (CDC) and increased tumor cell survival.

That CD46 isoforms may differ in their attenuation or promotion of bladder cancer also has been investigated (71). Using a xenograft model, CD46 CYT-1-isoforms attenuated while CYT-2 isoforms promoted cell growth, migration, and tumorigenicity (71). The findings of this and similar investigations encourage the further dissection of signaling pathways impacted by CD46 expression and their disruption as an avenue for cancer therapeutics.

From these and other reports, it is clear that CD46 plays multiple roles in the progression of cancer (5, 57). However, exact mechanisms defining such interactions are incompletely understood and continue to be elucidated. What is consistent, though, is that when cancers lead to aberrantly high expression of CD46, the traditional role of complement as an antitumor effector, especially in association with therapeutic cancer-targeting mAbs, can be disrupted (72, 73). Thus, CDC as well as antibody-dependent cell–mediated cytotoxicity mechanisms may be diminished or abrogated, impairing antitumor defenses (73).

It is also important to note that there can be variability in CD46 expression even among similar tumor types. This can be seen when reviewing the literature (5) as well as consulting online resources such as The Cancer Genome Atlas Program (https://portal.gdc.cancer.gov/genes/ENSG00000117335). For example, a report profiling tumor tissues in microarrays determined that more than 35% of patients with colon/prostate carcinoma assessed demonstrated CD46 upregulation, while in other types of cancers (e.g., lung, brain, lymphoma) less than 11% of tissue samples showed increased CD46 expression (74). These and other similar studies point to the potential value of utilizing CD46 as a diagnostic and prognostic indicator for certain cancer types (5, 6). They also highlight the likely importance of dissecting the role of CD46 in multiple settings in order to select the optimal patient cohorts who could profit from CD46-targeted therapeutics.

Studying the roles of CD46 in cancer biology is hampered, because mice, an often-used oncologic model system, as well as other subprimates have very restricted expression of CD46. Wild-type mice (and rodents in general) primarily express a *Cd46* gene on the inner acrosomal membrane of spermatozoa and in the eye (18, 75), although other limited locations are possible (e.g., CNS) (76). Thus, no small animal model system is yet available to probe in vivo roles beyond that of CD46-transgenic mice (77).

### CD46, the complosome, and noncanonical roles in cancer.

An exciting finding in recent complement biology is that most cells contain a complosome consisting of intracellularly residing complement components that not only assist the immune defenses in plasma, but also facilitate key interactions within host cells (32, 33). While early underpinnings of the complosome were discovered only slightly more than 10 years ago (28, 31), this young field continues to grow; components and mechanisms are being characterized and controversies are being addressed (32, 33).

We now know that, intriguingly, the complosome components help direct basic cellular physiological processes, such as cell metabolism (28), autophagy (78), and gene expression (32, 33, 79). Those processes could impact or be affected by malignant transformation. Intracellular complement components are spawned from the same genes that are responsible for the liver-derived circulating components (32). Similar to its roles in blood, intracellular C3 is a central complosome component. However, the intracellular C3 cleavage products, C3a and C3b, can be generated in a convertase-independent manner (31). Other complosome players may include the C3a receptor (C3aR), C5, C5a, C5a receptor (C5aR), FH (80), and specialized forms of CD59 (81, 82) (reviewed in refs. 32, 33). In addition, components, including properdin, factor B, factor D, and C4, are beginning to be explored (83).

With regard to CD46, studies have demonstrated that its autocrine activation via intracellularly generated C3b, the “C3b/CD46 axis,” plays a key role in nutrient uptake and enhances cellular metabolism in CD4^+^ T cells (ref. 28 and reviewed in ref. 57). Furthermore, CD46 interacts with Jagged1, a Notch family member, to mediate the regulation of Th1 cell activation (84). Notch also regulates oxidative phosphorylation and glycolysis in cancer cells (28). Thus, the role of CD46 as a metabolic driver points to its involvement in malignant transformation and/or cellular proliferation, especially because cancer cells are known to be highly glycolytic and to anaerobically metabolize glucose (85, 86).

These findings may be relevant to numerous types of cancers, not only because the complosome has been identified in many cell types, but also since CD46 is ubiquitously expressed on most all nucleated human cells. Intracellular C3 stores and “tonic” generation of intracellular C3a have been detected in monocytes, neutrophils, CD8^+^ T cells, B cells, and normal epidermal keratinocytes (refs. 31, 87 and reviewed in ref. 32). Furthermore, C3 is expressed intracellularly in donor human pancreatic islet β cells where it can regulate autophagy (78). In addition, islet C3 expression is upregulated in type two diabetes (78).

Thus, the almost universal nature of the complosome points to broad homeostatic functions for immune cells and a host of nonimmune cell types. Dysregulation of this system likely has important ramifications for cancer as well as for other diseases.

## CD46 targeting by therapeutic viral vectors

Two key characteristics make CD46 an attractive candidate for oncologic therapy. First, as outlined, it is overexpressed in a variety of malignant cells (Figure 3). Second, as also noted above, it is a receptor for several strains of AdVs and for the vaccine strain of MV that can be engineered for therapeutic applications (4–6). Consequently, modified viral vectors targeting CD46 currently are being exploited for a wide range of therapeutic applications, such as Ad26 vaccine vectors for treatment of HIV (88) and COVID-19 (89) and for multiple forms of cancer (see below).

### CD46-targeted oncolytic adenoviral therapy.

Oncolytic AdVs that specifically target CD46 represent an innovative approach in cancer treatment that leverages CD46 binding with the lytic and other capabilities of AdVs. Structurally, AdVs are nonenveloped, with double-stranded DNA genomes and an icosahedral capsid. The three major capsid proteins that may interact with cellular receptors are fiber, hexon, and penton (90, 91). Currently, at least 114 AdV serotypes have been identified that are classified into seven species (groups), A through G (92–94). Recombinant AdVs are probably the most commonly used viral vectors in gene therapies (95). One of the advantages of employing oncolytic adenoviral therapy is that, in addition to lysing cancer cells, the released tumor antigens may trigger a robust antitumor response, potentially initiating a more long-term response in certain cancers (37, 96).

Earlier studies often utilized AdVs of group C (Ad5) that target the coxsackie-AdV receptor (CAR) (92, 93, 96). However, two challenges limit the use of CAR-targeting AdVs. First, CAR expression on cancer cells can be intrinsically low or downregulated in certain tumors, leading to variable success (97, 98). Second, because Ad5 commonly causes upper respiratory tract and gastrointestinal infections, preexisting neutralizing antibodies to it are widespread in the population (97–99). This may prevent efficient therapeutic transduction of target cells (98, 99).

Alternatively, AdVs that engage CD46 may exploit its high expression on tumor cells, thereby enhancing therapeutic specificity and efficacy. Consequently, CD46-binding AdVs (such as some species of groups B and D) are being genetically modified to selectively infect and kill cancer cells and to reduce binding to normal cells (5, 90). Some therapeutic vectors are hybrids and consist of combinations of two AdVs such as may be generated by “directed evolution” (100) or by genetic engineering or both (90, 93).

Enadenotucirev (EnAd, formerly ColoAd1, see Table 1) is a novel AdV group B hybrid consisting of components from Ad3 and Ad11p, whose receptor binding is more potent than either of its parent viruses (100). CD46 was demonstrated to be a cellular receptor for Ad11p, the parent virus for EnAd that is a subgroup of Ad11 (40, 100, 101), although desmoglein-2 (DSG2) may also be a receptor (92, 102). EnAd mediates a nonapoptotic cell death via disrupting cellular membranes and release of proinflammatory mediators (103). It has been tested as a monotherapy in several clinical trials against a variety of cancers (NCT02053220, ref. 104; NCT02028442, ref. 105) and in combination with chemoradiotherapy (NCT03916510, ref. 106), chemotherapy (NCT02028117, ref. 107), and/or immunotherapy (NCT02636036, ref. 108) (Table 1). Overall, these studies demonstrate that i.v. administration of EnAd produces a manageable safety profile, stability in human blood, and an ability to increase tumor immune cell infiltration while specifically targeting cancer cells.

Next-generation versions of EnAd have also been engineered that introduce immunomodulating or other components as transgenes (Table 1). The variant, NG-350A, includes an agonistic anti-CD40 mAb for potential immunomodulation and neoplastic activities (NCT03852511, ref. 109, and NCT05165433). This has been assessed in trials of patients with metastatic/advanced epithelial tumors. Another variant of EnAd, NG-641, encodes four immunostimulatory transgenes: human fibroblast-activating protein–directed (FAP-directed) bispecific T cell activator antibody, IFN-α2, and CXCL9 and -10 (NCT04053283, NCT04830592, and NCT05043714) (110, 111).

Therapeutic vectors may also consist of two different AdV groups. A preclinical study using cancer cell lines as well as a murine xenograft model system compared a species C CAR-targeting Ad5 vector to a species B CD46-targeting Ad35 to create an Ad5/35 chimera. The CD46-targeted AdV chimera demonstrated significantly reduced tumor growth in both bladder and colorectal cancer models (74, 112).

Another Ad5/35 chimeric therapeutic, termed LOAd703 (delolimogene mupadenorepvec), has been developed and tested clinically. It also merges components of Ad5 spliced to the fiber and knob elements of Ad35 to confer CD46 targeting (113, 114). Additionally, LOAd703 is double-armed with two transgenes, a trimerized, membrane-bound CD40L and 4-1BB (also known as CD137L or TNFSF9), which are under the direction of a CMV promoter. These modifications are designed to confer immunostimulatory and antineoplastic activities (114). Clinical studies of LOAd703 in combination with other therapies are being conducted. Results of one phase I study (NCT02705196) concluded that the data demonstrate antitumor activity sufficient to warrant continuing the trials (114).

ICVB-1042 uses another AdV oncolytic combination, incorporating an engineered chimeric Ad5/Ad34 fiber that targets CD46 for entry into malignant cells (115). This rationally designed therapeutic contains numerous modifications to enhance viral replication, lysis, and spreading. Addition of a yellow fluorescent protein variant reporter permits an assessment of ICVB-1042 replication during lytic infection. Preclinical studies showed acceptable safety and toxicity profiles in murine models with effective control of tumors in both a bladder and a breast human xenograft cancer model (115). Phase I clinical studies of advanced solid tumors are being conducted (NCT05904236, see Table 1).

AdV therapeutic strategies continue to be developed. For example, instead of engaging the oncolytic properties of AdV vectors, Wang et al. developed a recombinant AdV35 fiber knob protein, Ad35K++, that can transiently remove CD46 from the cell surface for the purpose of sensitizing lymphoma cells to CDC killing triggered by the CD20-specific mAb, rituximab (116). The high affinity Ad35K++ cross-links several CD46 molecules on cancer cells, resulting in the shedding of CD46’s ectodomain and internalization of the remainder (116). The group also performed preclinical safety studies of Ad35K++, evaluating it as a novel rituximab cotherapeutic (117). Subsequently, a combination approach investigated the preclinical targeting of MM. The researchers found that Ad35K++ along with a peptide inhibitor of CD59 (a regulator of the membrane attack complex) effectively increased CDC triggered by addition of the MM therapeutic mAbs (daratumumab and isatuximab that target CD38) (118). Human clinical trials are anticipated.

### CD46-targeted MV oncolytic therapy.

Attenuated MV systems represent a second route for CD46-targeted oncolytic therapy (Table 2). MV is a negative-strand RNA paramyxovirus with six genes that encode eight proteins, of which hemagglutinin is the receptor attachment protein while the fusion protein facilitates interactions of the viral envelope with the host cell membrane (119, 120).

While wild-type MV is a serious infectious disease, attenuated versions such as the Edmonston (Edm) (vaccine) strain have excellent safety profiles (120, 121). CD46 is the receptor for the laboratory-grown MV-Edm (vaccine) strain (34, 35) whereas the wild-type MV targets SLAMF1 (CD150) on immune cells and nectin-4 (PVRL4) on epithelial cells (120). CD46 “retargeting” by MV occurred in cell culture as result of mutations in the MV hemagglutinin (attachment) protein (120). Note that MV-Edm attaches to CCP1 and -2 of CD46 (refs. 122, 123 and reviewed in ref. 4). Interestingly, New World primates exhibit a deletion of CCP1 of CD46 via alternative splicing, a change preventing binding and infection by MV (124).

The idea to utilize MV as a cancer treatment arose from earlier observations of tumor regression following MV infections (120, 125). For example, a well-cited case report described the remission of Burkitt’s lymphoma in a young boy following MV infection (120, 125). Thus, advantages for using MV-Edm as an oncolytic cancer therapy are its established safety profile, ability to replicate within and kill cancer cells, activation of antitumor responses, and suitability for genetic engineering (120, 126). Furthermore, MV-Edm is able to distinguish between high CD46 densities typical of tumor cells and lower CD46 densities characteristic of normal cells to promote the preferential killing of tumor cells (127). Thus, MV-Edm can exploit entry as well as cell-to-cell fusion for cytoreductive cancer treatments. Challenges include the possibility of preexisting neutralizing antibodies, the likely necessity for the tumor to overexpress CD46, and potential difficulty of manufacturing such agents (120).

A number of modified MV oncolytic vectors, though, have been developed and studied (Table 2). Galanis et al. tested an engineered MV-Edm strain that expresses the soluble extracellular domain of carcinoembryonic antigen (CEA) (125). Production of the CEA marker during viral replication provides safe quantitative monitoring of viral gene expression. MV-CEA was tested against ovarian cancer (NCT00408590) (125) and glioblastoma (NCT00390299) (126). These trials document the development of tumor-specific immune responses leading to antitumor effects and that the treatment was well tolerated (125, 126). The glioblastoma trial also demonstrated that the treatment was safe with repetitive intratumoral administration and was without a dose-limiting toxicity (126).

Another recombinant MV-Edm vector was generated by addition of the gene for human thyroidal sodium iodide symporter (NIS) (128). The NIS facilitates noninvasive monitoring and synergistically boosts oncolytic potency by the administration of iodine-131 that deposits a tissue-destructive dose of β radiation (128, 129). A phase I clinical trial (NCT00450814) also employing MV-NIS assessed the i.v. treatment of 32 patients with MM (129, 130). Overall, this investigation demonstrated a clear dose response with more sustained viremia at higher doses. These studies also concluded that the relative safety of the therapeutic agent coupled with one “dramatic” response and four transiently improved responses encourages further study and the addition of combination therapies such as immune checkpoint inhibitors (129, 130). A study to treat ovarian cancer also utilized MV-NIS (phase I/II, NCT02068794). The study established that the treatment triggered cellular immunity against the tumor, was well tolerated, and was associated with a promising median overall survival (NCT03171493) (131). Preliminary analysis of another investigation targeting bladder cancer found a higher-than-anticipated rate of tumor downstaging, suggesting that intravesical administration of MV-NIS has clinical utility against bladder cancer and may act synergistically with checkpoint blockade therapies (132). Clinical studies testing MV-NIS are active or completed against a variety of cancers, such as ovarian, breast, mesothelioma, medulloblastoma, and others (Table 2). Overall, MV-based virotherapies have demonstrated an acceptable safety profile, tumor selectivity, effective bystander killing effects, and an ability to manipulate the treatment via genetic engineering (119, 120, 126).

CD46-targeted MV technologies continue to be developed and refined. Because MV seropositivity can hinder utilization of MV anticancer therapies, a modified MV vaccine strain, MeV Stealth, is being developed that escapes anti-MV antibodies in vivo while maintaining its oncolytic properties (133). The approach may represent a potential alternative strategy to current MV oncolytic therapeutic agents (133).

## Antibody-drug conjugates

Another oncologic treatment approach employs a human monoclonal CD46-targeting antibody-drug conjugate (ADC) (Table 3) (designated FOR46) bearing a potent antimitotic agent, monomethyl auristatin E (MMAE) (61). The therapeutic followed a path of development in which a phage-isolated scFv, called UA20, was used to capture a tumor antigen identified as CD46 via mass spectrometry. Subsequently, a new human full-length IgG1 antibody, YS5 (also known as 23AG2), was developed. This antibody bound the same epitope on CD46 recognized by UA20, internalized via macropinocytosis (a relatively tumor-specific uptake mechanism), and demonstrated favorable developability for clinical translation (134–136).

FOR46 has been tested preclinically and utilized in several clinical trials (137–139) to treat MM and prostate cancer (Table 3). As noted earlier, relapsed MM often features up to a 14-fold increase in CD46 expression as a result of the genomic amplification of a segment on chromosome 1q that carries the *CD46* gene (61). The potential of therapeutic targeting using the CD46-ADC was demonstrated preclinically by its inhibition of myeloma cell proliferation in an orthometastatic xenograft (mouse) model (61). Additionally, preclinical drug efficacy for MM was established in a patient-derived xenograft model (140). A completed phase I trial in patients with relapsed or refractory MM found an acceptable toxicity profile and encouraging evidence of efficacy (NCT03650491) (137). Other studies of the CD46-ADC in a prostate cancer model system also demonstrated that it potently and selectively killed prostate cancer cell lines but not normal cells (136). Several clinical trials utilizing FOR46 to treat prostate cancer are now active or completed (138, 139) (Table 3). A multicenter single-agent trial (NCT03575819) determined that FOR46 (a) was well tolerated, (b) showed no evidence of CD46 on-target toxicity, and (c) provided evidence of efficacy in heavily pretreated patients (138). Thus, FOR46 has demonstrated clinical activity in patients with prostate cancer with an acceptable safety profile similar to other MMAE-containing ADCs (61, 138). This therapeutic is also being assessed in combination with enzalutamide, an anti-androgen (hormonal) therapeutic in a phase I/II trial targeting metastatic prostate cancer (NCT05011188) (139). Furthermore, Wang et al. engineered a radiopharmaceutical-labeled version of YS5, [^89^Zr]DFO-YS5, as a probe for PET. Zirconium-89 is the radioactive isotope while DFO (deferoxamine) is a chelator for ^89^Zr. The [^89^Zr]DFO-YS5 has been assessed preclinically as a potential imaging agent for both prostate cancer (141) and MM (142). Clinical trials are currently testing its utility as a theranostic agent and companion biomarker for prostate cancer (NCT05245006) and MM (NCT05892393). Finally, preclinical studies linking YS5 to other therapeutic agents are being undertaken; for example, two radioimmunotherapies employing YS5 linked to α particle emitters are being developed to treat prostate cancer: [^212^Pb]TCMC-YS5 (143) and [^225^Ac]DOTA-YS5 (144).

It will be of interest to see if other antibody-based approaches emerge that can offer tumor selectivity, a favorable safety profile, and, of course, efficacy.

## Conclusions

The future of CD46-targeted anticancer therapeutics, whether via oncolytic viruses, ADCs, or other modalities, holds considerable promise. The success of preclinical studies utilizing these technologies has already led to multiple clinical trials. Yet several challenges remain as summarized below. (a) The need continues for enhancing therapeutic effectiveness by decreasing the potential for provoked immune responses (preexisting antibodies) as well as improving patient stratification to address tumor heterogeneity of CD46 expression. (b) In addition, of paramount importance is assuring tumor specificity in order to reduce off-target effects for this widely expressed protein. To that end, numerous studies have demonstrated that increased expression levels of CD46 on cancer cells can assist in achieving therapeutic specificity. For example, tumor cells with high CD46 density were preferentially killed relative to lower expressing nontransformed cells by the oncolytic agent, MV-Edm (127). Additionally, in a MM model, Ong et al. found a correlation between the extent of cytopathic effects of cell fusion induced by MV and higher CD46 expression on malignant plasma cells whereas it was not cytotoxic to normal bone marrow progenitor cells (145). Furthermore, in a study utilizing both CD46-transgenic mice and macaques, transient depletion of CD46 was safe and well tolerated (146). Early and encouraging results of clinical trials such as with EnAD demonstrated that it was well-tolerated via i.v. infusion without serious cytokine release events (104). Moreover, an immunoPET probe, which targeted CD46 in vivo in several models of prostate cancer (141), demonstrated that the probe localized with specificity primarily to the CD46^+^ tumor. Indeed, multiple studies from ongoing clinical trials have reported primarily well-tolerated tumor-specific effects (Tables 1–3). (c) However, because CD46 plays many important roles, such as in reproductive health, T cell modulation, and cell metabolism, in-depth investigations will be needed to assess potential toxicities and verify safety in these and other realms of CD46-targeted therapeutics. (d) Perhaps the most promising avenue in the fight against cancer will be in utilizing CD46-targeted approaches in combination with other therapeutics, such as immune checkpoint inhibitors, chemotherapy, or radiotherapy. This, coupled with emerging innovations in genetic modifications and synthetic biology, could ultimately offer more effective, personalized, and safer treatment options for patients with cancer. CD46 may well become part of the established arsenal of key players in cancer therapeutics.

## Figures and Tables

**Table 3:**
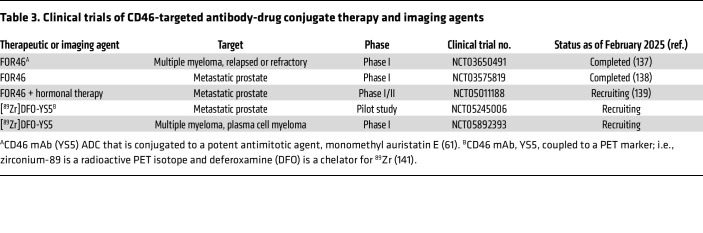
Clinical trials of CD46-targeted antibody-drug conjugate therapy and imaging agents

**Table 2:**
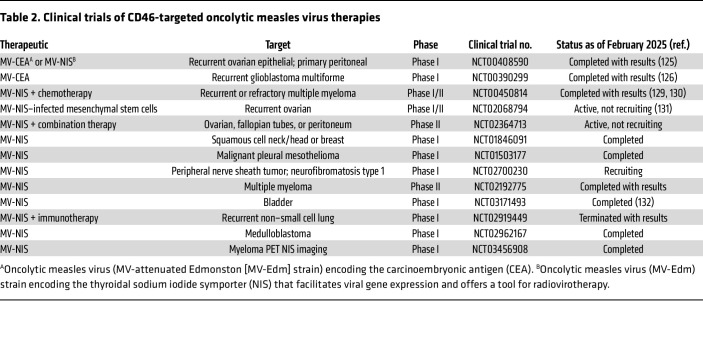
Clinical trials of CD46-targeted oncolytic measles virus therapies

**Table 1:**
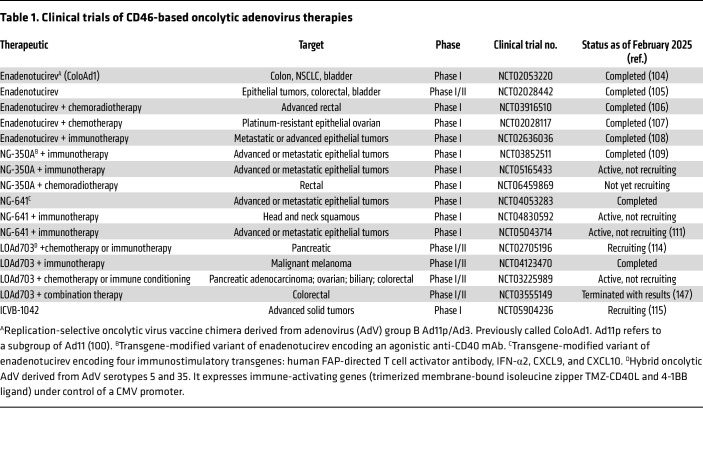
Clinical trials of CD46-based oncolytic adenovirus therapies

## Version 1 05/01/2025

Electronic publication

## References

[1] Cole JL (1985). Identification of an additional class of C3-binding membrane proteins of human peripheral blood leukocytes and cell lines. Proc Natl Acad Sci U S A.

[2] Seya T (1986). Purification and characterization of a membrane protein (gp45-70) that is a cofactor for cleavage of C3b and C4b. J Exp Med.

[3] Liszewski MK, Kemper C (2019). Complement in motion: the evolution of CD46 from a complement regulator to an orchestrator of normal cell physiology. J Immunol.

[4] Liszewski MK, Atkinson JP (2021). Membrane cofactor protein (MCP; CD46): deficiency states and pathogen connections. Curr Opin Immunol.

[5] Elvington M (2020). CD46 and oncologic interactions: friendly fire against cancer. Antibodies (Basel).

[6] Geller A, Yan J (2019). The role of membrane bound complement regulatory proteins in tumor development and cancer immunotherapy. Front Immunol.

[7] Kareem S (2023). Complement: Functions, location and implications. Immunology.

[8] Reis ES (2019). New insights into the immune functions of complement. Nat Rev Immunol.

[9] Forneris F (2016). Regulators of complement activity mediate inhibitory mechanisms through a common C3b-binding mode. EMBO J.

[10] Rodríguez de Córdoba S (1999). An integrated map of the human regulator of complement activation (RCA) gene cluster on 1q32. Mol Immunol.

[11] Liszewski MK (1991). Membrane cofactor protein (MCP or CD46): newest member of the regulators of complement activation gene cluster. Annu Rev Immunol.

[12] Fromell K (2020). Assessment of the role of C3(H_2_O) in the alternative pathway. Front Immunol.

[13] Ekdahl KN (2019). Is generation of C3(H_2_O) necessary for activation of the alternative pathway in real life?. Mol Immunol.

[14] Kojima A (1993). Membrane cofactor protein (CD46) protects cells predominantly from alternative complement pathway-mediated C3-fragment deposition and cytolysis. J Immunol.

[15] Barilla-LaBarca ML (2002). Role of membrane cofactor protein (CD46) in regulation of C4b and C3b deposited on cells. J Immunol.

[16] Liszewski MK, Atkinson JP (1996). Membrane cofactor protein (MCP; CD46). Isoforms differ in protection against the classical pathway of complement. J Immunol.

[17] Yamamoto H (2013). CD46: the ‘multitasker’ of complement proteins. Int J Biochem Cell Biol.

[18] Riley-Vargas RC (2004). CD46: expanding beyond complement regulation. Trends Immunol.

[19] Harris CL (2006). Complement and complement regulators in the male reproductive system. Mol Immunol.

[20] Lee SW (2002). CD46 is phosphorylated at tyrosine 354 upon infection of epithelial cells by Neisseria gonorrhoeae. J Cell Biol.

[21] Joubert PE (2009). Autophagy induction by the pathogen receptor CD46. Cell Host Microbe.

[22] Meiffren G (2010). Pathogen recognition by the cell surface receptor CD46 induces autophagy. Autophagy.

[23] Ludford-Menting MJ (2002). A functional interaction between CD46 and DLG4: a role for DLG4 in epithelial polarization. J Biol Chem.

[24] West EE (2018). Complement and the regulation of T cell responses. Annu Rev Immunol.

[25] Killick J (2018). Complement as a regulator of adaptive immunity. Semin Immunopathol.

[26] Cardone J (2010). Complement regulator CD46 temporally regulates cytokine production by conventional and unconventional T cells. Nat Immunol.

[27] Ni Choileain S (2011). The dynamic processing of CD46 intracellular domains provides a molecular rheostat for T cell activation. PLoS One.

[28] Kolev M (2015). Complement regulates nutrient influx and metabolic reprogramming during Th1 cell responses. Immunity.

[29] Arbore G (2018). Complement receptor CD46 co-stimulates optimal human CD8^+^ T cell effector function via fatty acid metabolism. Nat Commun.

[30] West EE (2020). Complement and human T cell metabolism: Location, location, location. Immunol Rev.

[31] Liszewski MK (2013). Intracellular complement activation sustains T cell homeostasis and mediates effector differentiation. Immunity.

[32] West EE, Kemper C (2023). Complosome - the intracellular complement system. Nat Rev Nephrol.

[33] King BC, Blom AM (2023). Intracellular complement: Evidence, definitions, controversies, and solutions. Immunol Rev.

[34] Naniche D (1993). Human membrane cofactor protein (CD46) acts as a cellular receptor for measles virus. J Virol.

[35] Dörig RE (1993). The human CD46 molecule is a receptor for measles virus (Edmonston strain). Cell.

[36] Gaggar A (2003). CD46 is a cellular receptor for group B adenoviruses. Nat Med.

[37] Persson BD (2021). Human species D adenovirus hexon capsid protein mediates cell entry through a direct interaction with CD46. Proc Natl Acad Sci U S A.

[38] Cattaneo R (2004). Four viruses, two bacteria, and one receptor: membrane cofactor protein (CD46) as pathogens’ magnet. J Virol.

[39] Marttila M (2005). CD46 is a cellular receptor for all species B adenoviruses except types 3 and 7. J Virol.

[40] Segerman A (2003). Adenovirus type 11 uses CD46 as a cellular receptor. J Virol.

[41] Wu E (2004). Membrane cofactor protein is a receptor for adenoviruses associated with epidemic keratoconjunctivitis. J Virol.

[42] Santoro F (1999). CD46 is a cellular receptor for human herpesvirus 6. Cell.

[43] Stein KR (2019). CD46 facilitates entry and dissemination of human cytomegalovirus. Nat Commun.

[44] Okada N (1995). Membrane cofactor protein (CD46) is a keratinocyte receptor for the M protein of the group A streptococcus. Proc Natl Acad Sci U S A.

[45] Källström H (1997). Membrane cofactor protein (MCP or CD46) is a cellular pilus receptor for pathogenic Neisseria. Mol Microbiol.

[46] Li K (2006). CD46 (membrane cofactor protein) acts as a human epithelial cell receptor for internalization of opsonized uropathogenic Escherichia coli. J Immunol.

[47] de Astorza B (2004). C3 promotes clearance of Klebsiella pneumoniae by A549 epithelial cells. Infect Immun.

[48] Mahtout H (2011). Fusobacterium nucleatum binding to complement regulatory protein CD46 modulates the expression and secretion of cytokines and matrix metalloproteinases by oral epithelial cells. J Periodontol.

[49] Li M (2022). Edwardsiella tarda TraT is an anti-complement factor and a cellular infection promoter. Commun Biol.

[50] Karp CL (1996). Mechanism of suppression of cell-mediated immunity by measles virus. Science.

[51] Kurita-Taniguchi M (2000). Functional modulation of human macrophages through CD46 (measles virus receptor): production of IL-12 p40 and nitric oxide in association with recruitment of protein-tyrosine phosphatase SHP-1 to CD46. J Immunol.

[52] Roumenina LT (2019). Context-dependent roles of complement in cancer. Nat Rev Cancer.

[53] Thurman JM (2020). Complement and cancer - A dysfunctional relationship?. Antibodies (Basel).

[54] Hussain N (2022). Targeting the complement system in pancreatic cancer drug resistance: a novel therapeutic approach. Cancer Drug Resist.

[55] Kleczko EK (2019). Targeting the complement pathway as a therapeutic strategy in lung cancer. Front Immunol.

[56] Meri S (2023). The Yin yang of complement and cancer. Cancer Immunol Res.

[57] Kolev M (2022). Inside-out of complement in cancer. Front Immunol.

[58] Zhang R (2019). Role of the complement system in the tumor microenvironment. Cancer Cell Int.

[59] Seya T (1990). Quantitative analysis of membrane cofactor protein (MCP) of complement. High expression of MCP on human leukemia cell lines, which is down-regulated during cell differentiation. J Immunol.

[60] Hara T (1992). Levels of complement regulatory proteins, CD35 (CR1), CD46 (MCP) and CD55 (DAF) in human haematological malignancies. Br J Haematol.

[61] Sherbenou DW (2016). Antibody-drug conjugate targeting CD46 eliminates multiple myeloma cells. J Clin Invest.

[62] Kinugasa N (1999). Expression of membrane cofactor protein (MCP, CD46) in human liver diseases. Br J Cancer.

[63] Seya T (1995). Purification and functional properties of soluble forms of membrane cofactor protein (CD46) of complement: identification of forms increased in cancer patients’ sera. Int Immunol.

[64] Hakulinen J (2004). Complement inhibitor membrane cofactor protein (MCP; CD46) is constitutively shed from cancer cell membranes in vesicles and converted by a metalloproteinase to a functionally active soluble form. Eur J Immunol.

[65] Yu JH (2024). The complement regulatory protein CD46 serves as a novel biomarker for cervical cancer diagnosis and prognosis evaluation. Front Immunol.

[66] Surowiak P (2006). CD46 expression is indicative of shorter revival-free survival for ovarian cancer patients. Anticancer Res.

[67] Maciejczyk A (2011). CD46 Expression is an unfavorable prognostic factor in breast cancer cases. Appl Immunohistochem Mol Morphol.

[68] Lu Z (2014). Bioinformatic analysis of the membrane cofactor protein CD46 and microRNA expression in hepatocellular carcinoma. Oncol Rep.

[69] Thi TN (2024). Complement regulatory protein CD46 promotes bladder cancer metastasis through activation of MMP9. Int J Oncol.

[70] Buettner R (2007). Activated signal transducers and activators of transcription 3 signaling induces CD46 expression and protects human cancer cells from complement-dependent cytotoxicity. Mol Cancer Res.

[71] Zeng J (2021). CD46 splice variant enhances translation of specific mRNAs linked to an aggressive tumor cell phenotype in bladder cancer. Mol Ther Nucleic Acids.

[72] Elvington M (2012). A targeted complement-dependent strategy to improve the outcome of mAb therapy, and characterization in a murine model of metastatic cancer. Blood.

[73] Macor P (2018). Complement as a biological tool to control tumor growth. Front Immunol.

[74] Cho YS (2016). Efficacy of CD46-targeting chimeric Ad5/35 adenoviral gene therapy for colorectal cancers. Oncotarget.

[75] Lyzogubov V (2014). Complement regulatory protein CD46 protects against choroidal neovascularization in mice. Am J Pathol.

[76] Esposito P (2023). CD46 expression in the central nervous system of male and female pubescent mice. J Neuroimmunol.

[77] Kemper C (2001). Membrane cofactor protein (MCP; CD46) expression in transgenic mice. Clin Exp Immunol.

[78] King BC (2019). Intracellular cytosolic complement component C3 regulates cytoprotective autophagy in pancreatic beta cells by interaction with ATG16L1. Autophagy.

[79] Kremlitzka M (2019). Interaction of serum-derived and internalized C3 with DNA in human B cells-A potential involvement in regulation of gene transcription. Front Immunol.

[80] Daugan MV (2021). Intracellular factor H drives tumor progression independently of the complement cascade. Cancer Immunol Res.

[81] Golec E (2019). A cryptic non-GPI-anchored cytosolic isoform of CD59 controls insulin exocytosis in pancreatic β-cells by interaction with SNARE proteins. FASEB J.

[82] Golec E (2022). Alternative splicing encodes functional intracellular CD59 isoforms that mediate insulin secretion and are down-regulated in diabetic islets. Proc Natl Acad Sci U S A.

[83] Liszewski MK (2017). Complement’s hidden arsenal: New insights and novel functions inside the cell. Mol Immunol.

[84] Le Friec G (2012). The CD46-Jagged1 interaction is critical for human TH1 immunity. Nat Immunol.

[85] Hess C, Kemper C (2016). Complement-mediated regulation of metabolism and basic cellular processes. Immunity.

[86] Bose S (2021). Glucose metabolism in cancer: the warburg effect and beyond. Adv Exp Med Biol.

[87] Mommert S (2021). C3a and its receptor C3aR are detectable in normal human epidermal keratinocytes and are differentially regulated via TLR3 and LL37. J Innate Immun.

[88] Stieh DJ (2023). Safety and immunogenicity of Ad26-Vectored HIV vaccine with mosaic immunogens and a novel mosaic envelope protein in HIV-uninfected adults: a phase 1/2a Study. J Infect Dis.

[89] Magaret CA (2024). Quantifying how single dose Ad26.COV2.S vaccine efficacy depends on Spike sequence features. Nat Commun.

[90] Barry MA (2020). Retargeting adenoviruses for therapeutic applications and vaccines. FEBS Lett.

[91] Watanabe M (2021). Adenovirus biology, recombinant adenovirus, and adenovirus usage in gene therapy. Viruses.

[92] MacNeil KM (2023). Adenoviruses in medicine: innocuous pathogen, predator, or partner. Trends Mol Med.

[93] Gryciuk A (2023). Oncolytic adenoviruses armed with co-stimulatory molecules for cancer treatment. Cancers (Basel).

[94] Scarsella L (2024). Advances of recombinant adenoviral vectors in preclinical and clinical applications. Viruses.

[95] Wallace R (2024). The immune system-a double-edged sword for adenovirus-based therapies. Viruses.

[96] Oronsky B (2022). Oncolytic adenoviruses: the cold war against cancer finally turns hot. Cancers (Basel).

[97] Hulin-Curtis SL (2016). Evaluation of CD46 re-targeted adenoviral vectors for clinical ovarian cancer intraperitoneal therapy. Cancer Gene Ther.

[98] Ono R (2023). Treatment of human pancreatic cancers following local and systemic administration of oncolytic adenovirus Serotype 35. Anticancer Res.

[99] Wang H (2023). HDAd6/35++ - A new helper-dependent adenovirus vector platform for in vivo transduction of hematopoietic stem cells. Mol Ther Methods Clin Dev.

[100] Kuhn I (2008). Directed evolution generates a novel oncolytic virus for the treatment of colon cancer. PLoS One.

[101] Fleischli C (2007). Species B adenovirus serotypes 3, 7, 11 and 35 share similar binding sites on the membrane cofactor protein CD46 receptor. J Gen Virol.

[102] Wang H (2011). Desmoglein 2 is a receptor for adenovirus serotypes 3, 7, 11 and 14. Nat Med.

[103] Dyer A (2017). Oncolytic group B adenovirus enadenotucirev mediates non-apoptotic cell death with membrane disruption and release of inflammatory mediators. Mol Ther Oncolytics.

[104] Garcia-Carbonero R (2017). Phase 1 study of intravenous administration of the chimeric adenovirus enadenotucirev in patients undergoing primary tumor resection. J Immunother Cancer.

[105] Machiels JP (2019). A phase 1 dose escalation study of the oncolytic adenovirus enadenotucirev, administered intravenously to patients with epithelial solid tumors (EVOLVE). J Immunother Cancer.

[106] O’Cathail SM (2020). A phase 1 trial of the safety, tolerability and biological effects of intravenous Enadenotucirev, a novel oncolytic virus, in combination with chemoradiotherapy in locally advanced rectal cancer (CEDAR). Radiat Oncol.

[107] Moreno V (2021). Safety and efficacy of the tumor-selective adenovirus enadenotucirev with or without paclitaxel in platinum-resistant ovarian cancer: a phase 1 clinical trial. J Immunother Cancer.

[108] Fakih M (2023). Safety and efficacy of the tumor-selective adenovirus enadenotucirev, in combination with nivolumab, in patients with advanced/metastatic epithelial cancer: a phase I clinical trial (SPICE). J Immunother Cancer.

[109] Patel M (2023). Phase 1 clinical trial results for NG-350A, a novel transgene-armed and tumor-selective vector: Differential effects of intravenous (IV) versus intratumoral (IT) dosing on immune pharmacodynamics (PD). J Clin Oncol.

[110] Khalil DN (2023). A tumor-selective adenoviral vector platform induces transient antiphospholipid antibodies, without increased risk of thrombosis, in phase 1 clinical studies. Invest New Drugs.

[111] Simon G (2022). 762 First-in-human phase 1a study of NG-641, a tumour-selective vector expressing a FAP-TAc bispecific antibody and immune enhancer module, in patients with metastatic/advanced epithelial tumours (STAR). J Immunotherap Cancer.

[112] Do MH (2018). Targeting CD46 enhances anti-tumoral activity of adenovirus type 5 for bladder cancer. Int J Mol Sci.

[113] Eriksson E (2017). Shaping the tumor stroma and sparking immune activation by CD40 and 4-1BB signaling induced by an armed oncolytic virus. Clin Cancer Res.

[114] Musher BL (2024). LOAd703, an oncolytic virus-based immunostimulatory gene therapy, combined with chemotherapy for unresectable or metastatic pancreatic cancer (LOKON001): results from arm 1 of a non-randomised, single-centre, phase 1/2 study. Lancet Oncol.

[115] Kato Y (2024). Nonclinical characterization of ICVB-1042 as a selective oncolytic adenovirus for solid tumor treatment. Commun Biol.

[116] Wang H (2010). A recombinant adenovirus type 35 fiber knob protein sensitizes lymphoma cells to rituximab therapy. Blood.

[117] Richter M (2016). Preclinical safety, pharmacokinetics, pharmacodynamics, and biodistribution studies with Ad35K++ protein: a novel rituximab cotherapeutic. Mol Ther Methods Clin Dev.

[118] Wang H (2024). CD46 and CD59 inhibitors enhance complement-dependent cytotoxicity of anti-CD38 monoclonal antibodies daratumumab and isatuximab in multiple myeloma and other B-cell malignancy cells. Cancer Biol Ther.

[119] Msaouel P (2018). Clinical trials with oncolytic measles virus: current status and future prospects. Curr Cancer Drug Targets.

[120] Engeland CE, Ungerechts G (2021). Measles virus as an oncolytic immunotherapy. Cancers (Basel).

[121] Gastañaduy PA (2021). Measles in the 21st century: progress toward achieving and sustaining elimination. J Infect Dis.

[122] Manchester M (1995). Measles virus and C3 binding sites are distinct on membrane cofactor protein (CD46). Proc Natl Acad Sci U S A.

[123] Manchester M (1997). Measles virus recognizes its receptor, CD46, via two distinct binding domains within SCR1-2. Virology.

[124] Hsu EC (1997). Artificial mutations and natural variations in the CD46 molecules from human and monkey cells define regions important for measles virus binding. J Virol.

[125] Galanis E (2010). Phase I trial of intraperitoneal administration of an oncolytic measles virus strain engineered to express carcinoembryonic antigen for recurrent ovarian cancer. Cancer Res.

[126] Galanis E (2024). Carcinoembryonic antigen-expressing oncolytic measles virus derivative in recurrent glioblastoma: a phase 1 trial. Nat Commun.

[127] Anderson BD (2004). High CD46 receptor density determines preferential killing of tumor cells by oncolytic measles virus. Cancer Res.

[128] Dingli D (2004). Image-guided radiovirotherapy for multiple myeloma using a recombinant measles virus expressing the thyroidal sodium iodide symporter. Blood.

[129] Russell SJ (2014). Remission of disseminated cancer after systemic oncolytic virotherapy. Mayo Clin Proc.

[130] Dispenzieri A (2017). Phase I trial of systemic administration of Edmonston strain of measles virus genetically engineered to express the sodium iodide symporter in patients with recurrent or refractory multiple myeloma. Leukemia.

[131] Galanis E (2015). Oncolytic measles virus expressing the sodium iodide symporter to treat drug-resistant ovarian cancer. Cancer Res.

[132] Naik S (2022). Safety and efficacy of neoadjuvant intravesical oncolytic MV-NIS in patients with urothelial carcinoma. J Clin Oncol.

[133] Muñoz-Alía M (2021). MeV-Stealth: A CD46-specific oncolytic measles virus resistant to neutralization by measles-immune human serum. PLoS Pathog.

[134] He J (2010). Targeting prostate cancer cells in vivo using a rapidly internalizing novel human single-chain antibody fragment. J Nucl Med.

[135] Commisso C (2013). Macropinocytosis of protein is an amino acid supply route in Ras-transformed cells. Nature.

[136] Su Y (2018). Targeting CD46 for both adenocarcinoma and neuroendocrine prostate cancer. JCI Insight.

[137] Wong S (2021). P-225: A first-in-human study of FOR46 in patients with triple refractory Multiple Myeloma. Clin Lymphoma Myeloma Leuk.

[138] Aggarwal RR (2022). Phase 1a/1b study of FOR46, an antibody drug conjugate (ADC), targeting CD46 in metastatic castration-resistant prostate cancer (mCRPC). J Clin Oncol.

[139] Shakhnazaryan N (2024). A phase 1b dose escalation study of FOR46, a novel antibody-drug conjugate targeting a tumor-specific epitope of CD46, in combination with enzalutamide (Enza) in patients with metastatic castration resistant prostate cancer (mCRPC). J Clin Oncol.

[140] VanWyngarden MJ (2023). CD46-ADC reduces the engraftment of multiple myeloma patient-derived xenografts. Cancers (Basel).

[141] Wang S (2021). Molecular imaging of prostate cancer targeting CD46 using ImmunoPET. Clin Cancer Res.

[142] Wadhwa A (2024). CD46-targeted theranostics for PET and 225Ac-radiopharmaceutical therapy of multiple myeloma. Clin Cancer Res.

[143] Li J (2023). CD46 targeted ^212^Pb alpha particle radioimmunotherapy for prostate cancer treatment. J Exp Clin Cancer Res.

[144] Bidkar AP (2023). Treatment of prostate cancer with CD46-targeted 225Ac alpha particle radioimmunotherapy. Clin Cancer Res.

[145] Ong HT (2006). Oncolytic measles virus targets high CD46 expression on multiple myeloma cells. Exp Hematol.

[146] Beyer I (2013). Transient removal of CD46 is safe and increases B-cell depletion by rituximab in CD46 transgenic mice and macaques. Mol Ther.

[147] Li C (2022). Hybrid-control arm construction using historical trial data for an early-phase, randomized controlled trial in metastatic colorectal cancer. Commun Med (Lond).

